# Evaluation of Composites Reinforced by Processed and Unprocessed Coconut Husk Powder

**DOI:** 10.3390/polym15051195

**Published:** 2023-02-27

**Authors:** David Coverdale Rangel Velasco, Felipe Perissé Duarte Lopes, Djalma Souza, Henry Alonso Colorado Lopera, Sergio Neves Monteiro, Carlos Maurício Fontes Vieira

**Affiliations:** 1 Advanced Materials Laboratory, State University of the North of Rio de Janeiro Darcy Ribeiro—UENF, Av Alberto Lamego, 2000, Campos dos Goytacazes CEP 28013-602, RJ, Brazil; 2 CCComposites, Engineering school, Universidad de Antioquia (UdeA), Medellín 050001, Colombia

**Keywords:** natural fiber, industrial agro waste, izod impact testing, coconut husk, polymeric composites

## Abstract

Engineering activities aim to satisfy the demands of society. Not only should the economic and technological aspects be considered, but also the socio-environmental impact. In this sense, the development of composites with the incorporation of waste has been highlighted, aiming not only for better and/or cheaper materials, but also optimizing the use of natural resources. To obtain better results using industrial agro waste, we need to treat this waste to incorporate engineered composites and obtain the optimal results for each application desired. The objective of this work is to compare the effect of processing coconut husk particulates on the mechanical and thermal behavior of epoxy matrix composites, since we will need a smooth composite in the near future to be applied by brushes and sprayers with a high quality surface finish. This processing was carried out in a ball mill for 24 h. The matrix was a Bisphenol A diglycidyl ether (DGEBA)/triethylenetetramine (TETA) epoxy system. The tests that were performed were resistance to impact and compression, as well as the linear expansion test. Through this work, it can be observed that the processing of coconut husk powder was beneficial, allowing not only positive improvements to the properties of the composite, but also a better workability and wettability of the particulates, which was attributed to the change in the average size and shape of particulates. That means that the composites with processed coconut husk powders have improved impact strength (46 up to 51%) and compressive strength (88 up to 334%), in comparison with unprocessed particles.

## 1. Introduction

**Figure 1:**
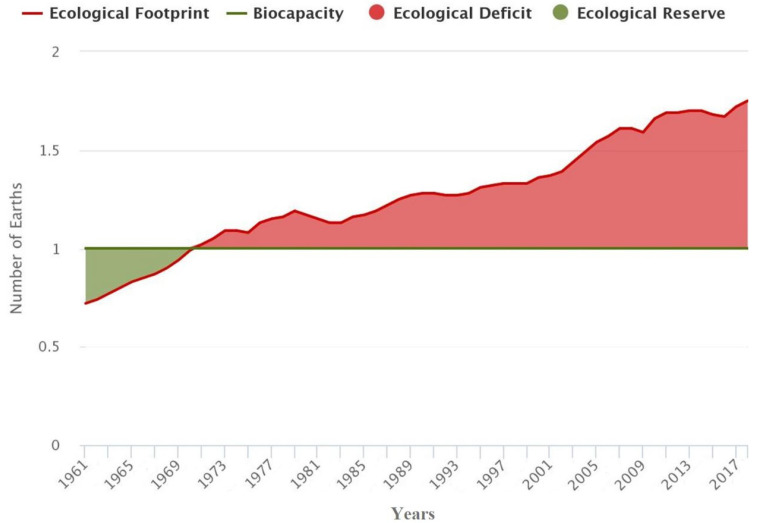
Global resource demand by year [1].

The population and the demand for resources have been growing annually. It is estimated that currently it takes a planet with 75% plus resources than the existing ones to sustain current human activities. That is, 1.75 planets (Figure 1). Furthermore, the more developed regions, such as Europe and North America, have an even bigger demand, 3 and 5.1 planets, respectively [1].

In addition, it is observed that countries with big energy resources, for example, tend to have a higher quality and expectancy of life [2]. In this vein, the Energy Information Administration estimates that the demand for new resources will increase by about 50% in 30 years [3].

For sustainable research development, humanity has sought alternative energy sources, ways of reusing energy, as well as ways of minimizing energy consumption in all fields, especially in the manufacturing industries. So, natural fibers that lost ground in the last century to synthetic fibers have gained relevance again for reasons such as this and also to have a green solution for new materials, developing ecofriendly resources [4,5,6,7].

A good example is the work of Wu [8] who evaluated the replacement of glass fibers by kenaf fibers in laminates. In this work, it was possible to observe not only a 33% reduction in the energy demand of the process, but also improved material properties.

In addition to energy consumption, Azevedo et al. [9] highlighted the two biggest challenges of this century, to reduce the use of synthetic raw materials and inappropriate waste disposal. In this sense, the use of waste in composites has gained prominence in the last few decades [10]. Agro-industrial residues have the potential to be used as raw material for energy production, as shown in the works of [11,12].

Coconut production, for example, is carried out in more than 90 countries, with an estimated total value of 63 million tons per year [13]. The main solid residue from the coconut industry is the coconut husk, with the fibers having the best uses in other fields and with a greater added value [14]. It is estimated that 1 million tons of coconut fibers are produced annually [15].

However, it is observed that such residue is underutilized. Brazil, for example, is the fifth largest producer of coconuts in the world, has a tradition in the cultivation of vegetable fibers, as well as expertise and technology in the sector, but only 10% of the total volume of coconut biomass is used [14]. Therefore, the particles released during the fibers’ beneficiation are neglected and nowadays have either landfill or being burned as fuel as their final destination.

Currently, there is research investigating coconut biomass for fuels, acid solutions, agricultural substrates, constituents in composites, etc. [16,17,18,19,20,21,22]. However, in the development of composites, although coconut fibers still have a prominent role, there is an increasing amount of research into the use of coconut waste in composites [23].

However, for the particles released to avoid dispersed dimensions, they require treatment before their use as a raw material in engineered composites [10]. In particular, this applies when we need a surface finish with a high quality; for example, when used as a filler or pigment in coatings. Some researchers are developing coatings with pyrolyzed carbon from waste tires for automotive coatings [24,25], whilst others are modifying the fiber surface using gamma radiation or coating the fibers before adding on polymers to have a better interface, which has presented improvements in quality [26,27,28,29].

The processing of particulates affects their size and/or shape, and this situation may change the properties, which has been a research subject in several studies [30,31].

In this sense, this work aims to characterize the behavior of composites reinforced with coconut husk powder in two configurations: unprocessed and processed under ball milling; to reduce the particle sizes and obtain homogeneity in the particles; with the aim in the near future of application for coatings, so for that purpose we need a very good and at least smooth surface finishing.

## 2. Materials and Methods

**Figure 2:**
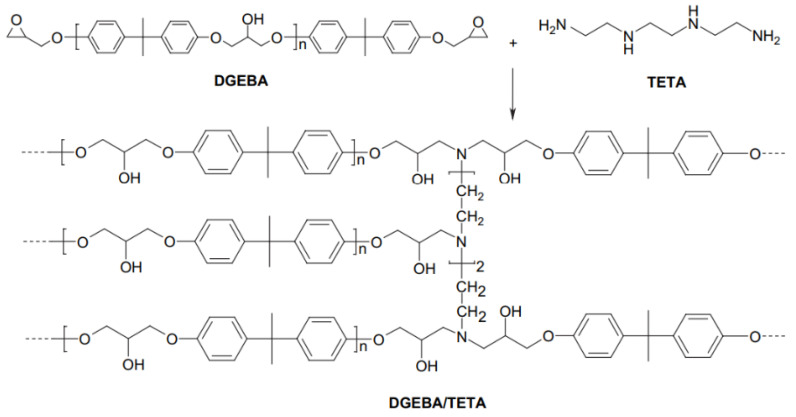
Schematic representation of DGEBA/TETA system [32].

The matrix used in this work was a two-component epoxy system, composed of Bisphenol A diglycidyl ether (DGEBA) resin and triethylene-tetramine (TETA) hardener, schematically shown in Figure 2 and sold, respectively, under the names SQ 2050 and SQ 3131 through Silaex Química Ltda, São Paulo, Brazil. This system has a medium viscosity, a rigid behavior and cures at room temperature.

**Figure 3:**
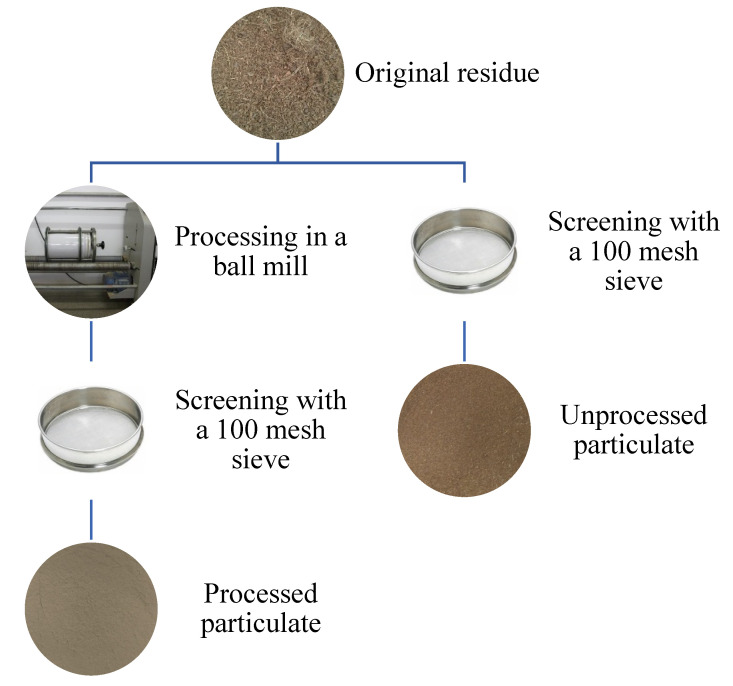
Scheme of particulate processing used in this work.

Figure 3 shows the difference between the process treatment and unprocessed coconut particulates. The unprocessed material was only sieved, in order to remove the large particles and larger fibers, using a sieve with an opening of 0.15 mm and 100 mesh. The material that was processed, before being sieved, was ground for 24 h in a ball mill, obtained from Servitech, model CT-240/A. This implies that the two materials used in this work, despite having passed through the same sieve, have a significant difference in average size and morphology. After sieving, the particulates’ morphology and dimensions were analyzed by Scanning Electron Microscopy (SEM). For this, the Shimadzu Superscan microscope model SSX-S50 was used, available at the Advanced Materials Laboratory (LAMAV) of the Universidade Estadual do Norte Fluminense Darcy Ribeiro (UENF).

The resin/hardener ratio used in this work was the one recommended by the manufacturer, 20 phr [33]. The filler amounts used were 10% and 20% of the volume for processed particulates and 10%, 20%, 30% and 40% for unprocessed particulate composites, in addition to a reference formulation of 0%. The maximum values used as a reference in the definition of such formulations were determined using as a reference the maximum amount of particulates in which it was possible to incorporate into the matrix keeping the mixture workable as a paste.

**Figure 4:**
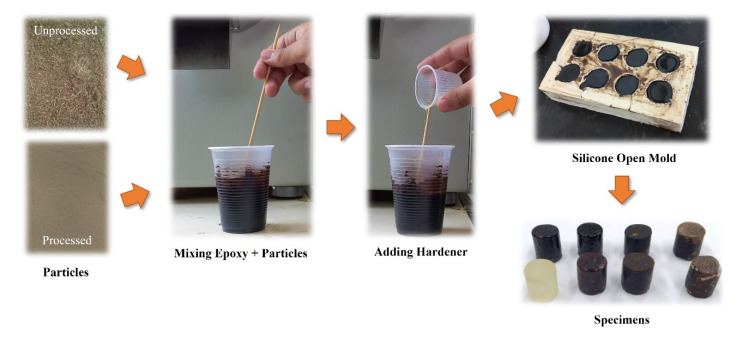
Specimens’ manufacture sequence.

The specimens were made in silicone open molds, as per the sequence shown in Figure 4, mixing first the coconut husk powders on epoxy manually for around 5 min or until the homogeneity was obtained; after that, the hardener was added into the mixture and poured into the silicone open molds.

The specimens were submitted to the tests listed in Table 1. In the table, one can also observe the number of specimens tested for each formulation, parameters adopted, standards used as reference as well as the machines used.

To verify if there was a statistically significant difference between the results of particles addition on mechanical properties, an analysis of variance (ANOVA) was performed, as well as the Tukey test. Such analyses were performed using the PAST software, and the Copenhagen and Holland algorithm [37], considering a significance level of 5%.

The ANOVA analysis consists of calculating the sum of squares between samples (Equation (1)) and within samples (Equation (2)) and dividing by the respective freedom degrees (Equations (3) and (4)) to find the variances (Equations (5) and (6)). The ratio of the between-sample and within-sample variance is called the F-ratio (Equation (7)) and is compared to critical values for a given chance α of concluding that there is a difference between groups when, in fact, there is not [38]:

(1) $$SQ_{entre}=\sum_{j=1}^{k}n_{j}{\left(\bar{X_{j}}-\overset{=}{X}\right)}^{2}$$

(2) $$SQ_{dentro}=\sum_{j=1}^{k}\sum_{i=1}^{n_{j}}{\left(X_{ij}-\bar{X_{j}}\right)}^{2}$$

(3) $$gl_{entre}=k-1$$

(4) $$gl_{dentro}=n-k$$

(5) $$S_{entre}^{2}=\frac{SQ_{entre}}{gl_{entre}}$$

(6) $$S_{dentro}^{2}=\frac{SQ_{dentro}}{gl_{dentro}}$$

(7) $$R_{F}=\frac{S_{entre}^{2}}{S_{dentro}^{2}}$$

Tukey’s test compares all possible pairs of means and is based on the Minimum Significant Difference (MSD), considering the group’s percentiles. In the calculation of the MSD, data extracted from the ANOVA were used to obtain the results.

## 3. Results and Discussion

**Figure 5:**
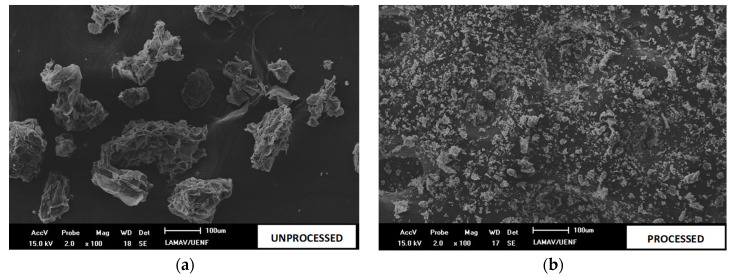
Coconut husk powder particles passed through a 100 mesh sieve: (**a**) unprocessed; (**b**) processed.

The micrographs of coconut shell particulates before and after processing can be seen in Figure 5a,b, both at 100× magnification. Through these, it is possible to observe that the processing resulted in a significant reduction in the average size of the particles, making it possible to pass through the sieve with 100 mesh (0.149 mm).

**Figure 6:**
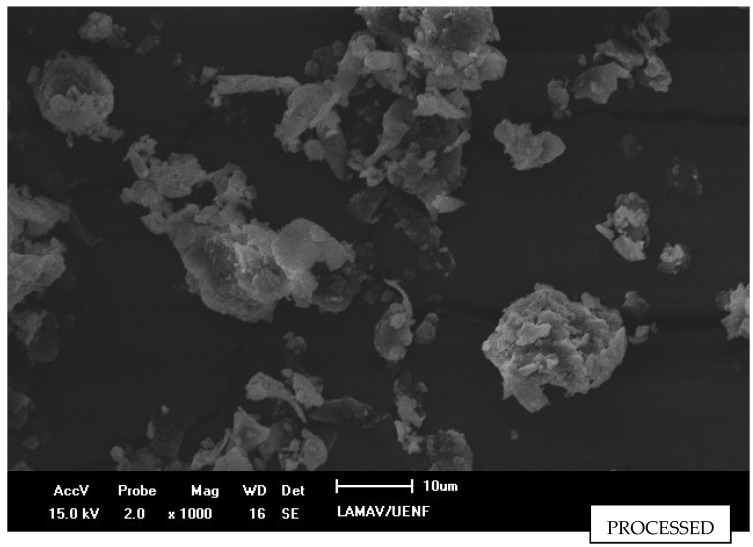
Processed particles after sieving on 100 mesh.

Figure 6 presents an image with a higher magnification of the coconut husk particulates, in which it is possible to observe that the particulates had a significant reduction in the average size. Due to this size reduction, the particulates tend to agglomerate. However, the aspect ratio tends to be reduced, as the short fibers originally present in the particulate tend to reduce the expectorate ratio. The morphology is more rounded after the milling process with a big dispersion in diameter below 15 µm.

Some researchers [39,40,41,42] have shown that composites with spherical particulates in a different matrix, such as ceramic, cement and polymers, improve the gain in workability and viscosity; corroborating that our processed particulates are a positive addition to the composites. Another benefit of spherical particulates is that it allows for the addition of more volume of particulate into the composite without losing the workability.

## 4. Compressive Testing

Table 2 presents the results obtained through the compression test. It is possible to observe the Young’s Modulus (YM), Yield Strength (YS) and Compressive Strength (CS), as well as the respective standard deviations of these properties. The latter, in general, are inferior in the formulations that use processed coconut husk powder.

**Figure 7:**
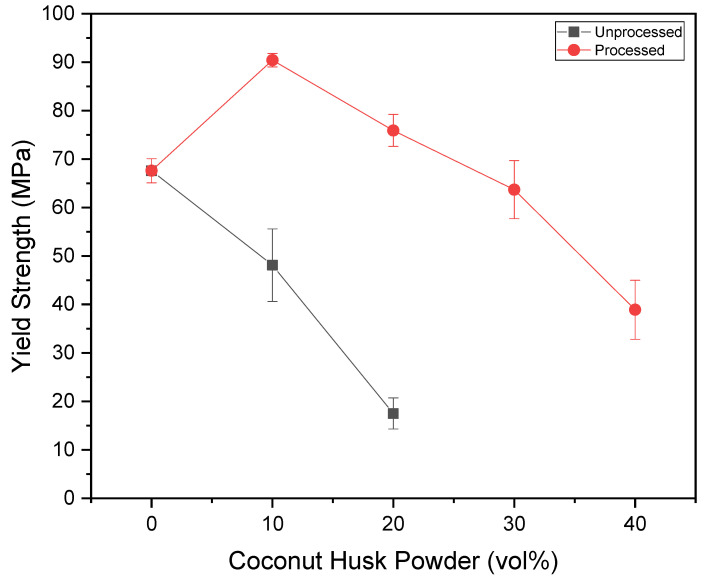
Variation of the yield strength versus coconut husk volume.

**Figure 8:**
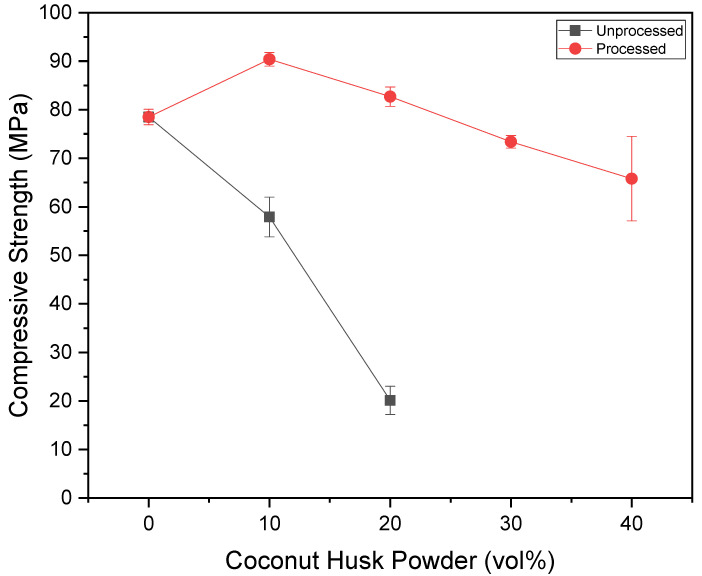
Variation of the compressive strength versus coconut husk volume.

Figure 7 and Figure 8 present the YS and CS results. It was possible to observe that the particulates’ processing was beneficial for the compressive strength of the composites, and was indicated by the ANOVA as a significant improvement. In addition, in formulations with 10% and 20% of processed filler, better results can be obtained than with pure epoxy, as well as the formulation with 30% results being close to the same. However, no formulation with unprocessed particulates has demonstrated this ability, as they have significantly lower resistance.

That means the composites with a lower size of particulates interfere in the damage mechanism blocking the cracks or creating a more broken surface area pushing the cracks to contour around the particle; using this behavior, we improved the strength of composites.

Sienckewiecks et al. (2022) [43] showed the natural materials used as fillers as a potential modifying agent for epoxy compositions, proving that bio-fillers of natural origin (such as coconut shell powder, cashew nut powder, hemp fibers, bamboo fibers, palm ash, waste tea fibers, banana bark fibers, rice husks, date seeds, pineapple leaves and others) positively change and improve the performance in epoxy composites.

Even if we change the matrix in composites, the behavior is almost the same, as observed by Sallal (2014) [44] using coconut shell powders reinforcing polyurethane resin; showing that with small particulates, the compressive strength increases and then reaches the maximum point creating a peak and afterwards decreasing with the addition of more powders.

**Figure 9:**
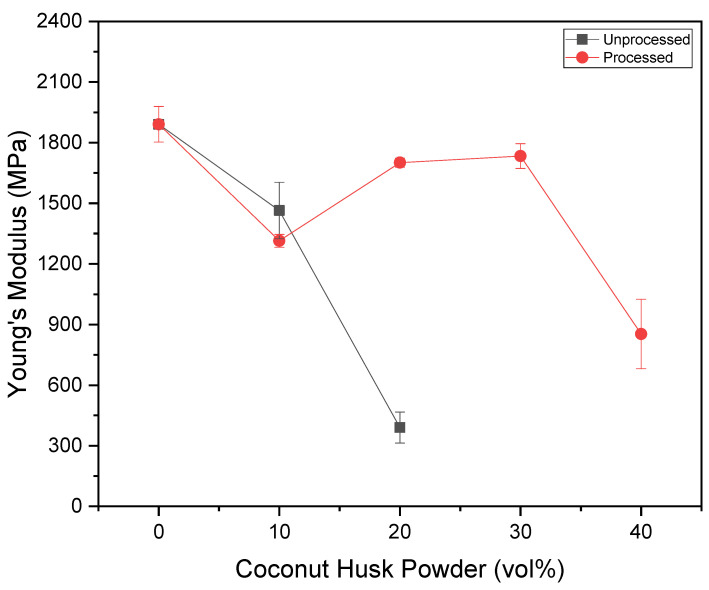
Variation of the Young’s modulus versus coconut husk powder.

Figure 9 shows the elastic modulus of composite materials. Then, it can be observed that formulations with intermediate load values present greater rigidity. On the other hand, formulations with load values close to 25% of the material incorporation capacity show intermediate stiffness values that are still higher than those of composites with the maximum amount of particulates. These, due to the high amount of particulates, present points with different levels of particulate concentration that impair the strength of the material and reduce its rigidity. ANOVA indicated that, in general, formulations with processed particulates have greater resistance, with an exception being the 10% formulation, which has no significant difference between formulations using processed and unprocessed material.

These behaviors were expected since the coconut husk powder is a porous material because of the lumen present in natural fibers, so this type of composite becomes soft, deforming more than the pure epoxy in the elastic region.

**Figure 10:**
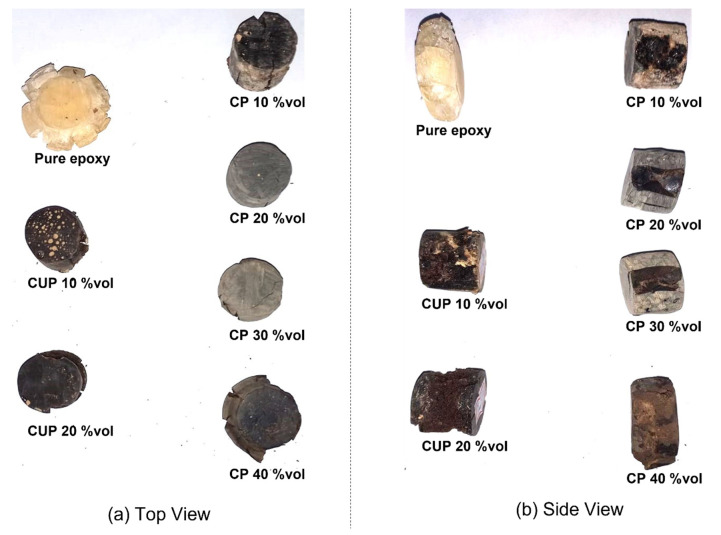
Specimen after compressive testing: (**a**) top view and (**b**) side view.

In Figure 10, the fracture aspects after compressive testing are shown; Figure 10a is the top view, where it is possible to observe that the pure epoxy is complete smashed, since there is no filler to block the polymer mobility, which is possible to see in side view in Figure 10b; the CUP 20 vol% achieves the lowest strength in all formulations, and the damages are concentrated in the middle and on several 45° cleavage planes, which are more evident in Figure 10b. For CP formulations, it is clear that until 30 vol% is attained, the deformation is uniformly provided by the compression load but maintains restraint of the total deformation and high values of the compression strength; the CP with 40 vol% is shown in Figure 10b, whereby the compression results in a larger deformation that is closer to pure epoxy. These behaviors were expected, since when we add particulate materials this creates a number of defects and then creates cracks which are a preferential way of developing into specimen until it breaks.

## 5. Impact Testing

Table 3 shows the results from the Impact Izod testing, obtaining the impact strength and notch strength.

**Figure 11:**
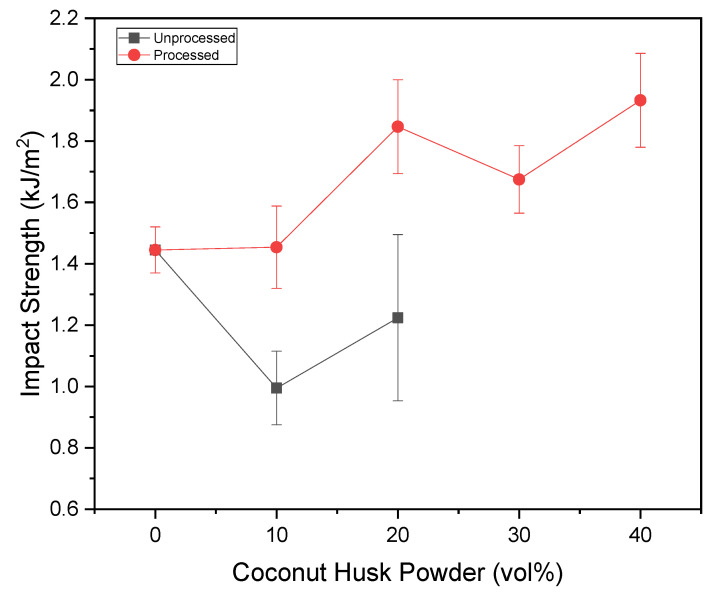
Impact Izod tests results versus coconut husk-powder volume amount.

**Figure 12:**
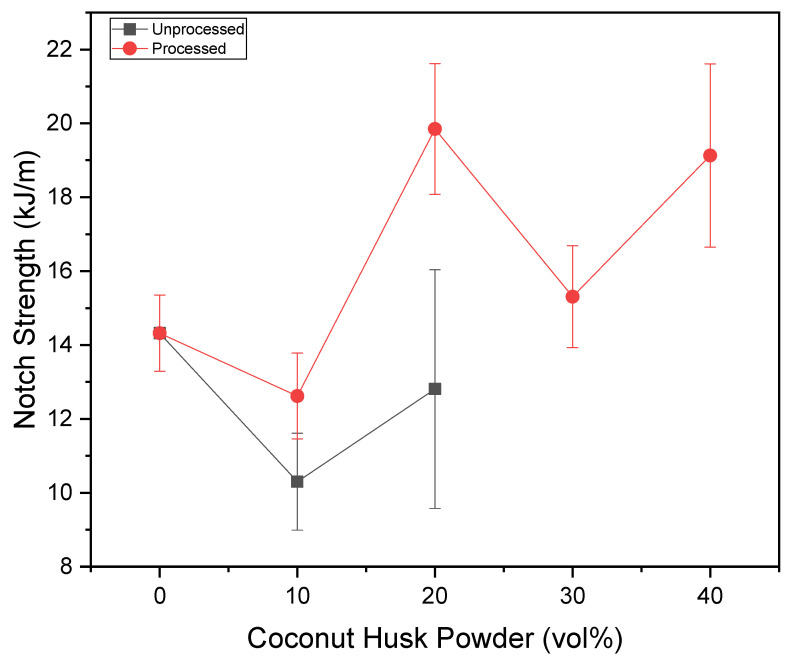
Impact Izod tests results versus coconut husk-powder volume amount.

Figure 11 and Figure 12 present the results in a graphic of the impact tests, in kJ/m^2^ for the impact strength and kJ/m for the notch strength. It is possible to observe that the processing of coconut husk powders is beneficial for the impact resistance of composites. Since the addition of unprocessed particulates significantly reduces the strength of composites and the addition of processed particulates significantly improves their strength. The exception observed was the formulation with 10% processed particulates; according to ANOVA, there is no significant resistance variation.

The same behavior for this type of composites was found by Andezai et al. (2020) [45], comparing coconut shell powder with two diameters, 0.150 mm and 0.212 mm; reinforcing the epoxy resin.

**Figure 13:**
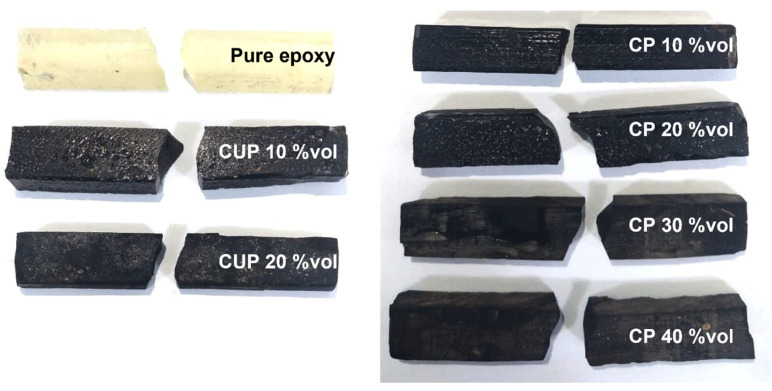
Specimen after impact testing.

About the rupture of the specimens submitted to the impact test, Figure 13 presents an example of each material tested, with the abbreviations CUP and CP representing Unprocessed Load and Processed Load, respectively. It is possible to observe that even with the addition of particulates all composites showed a fragile fracture mechanism.

## 6. Thermal Linear Dilatometry Testing

**Figure 14:**
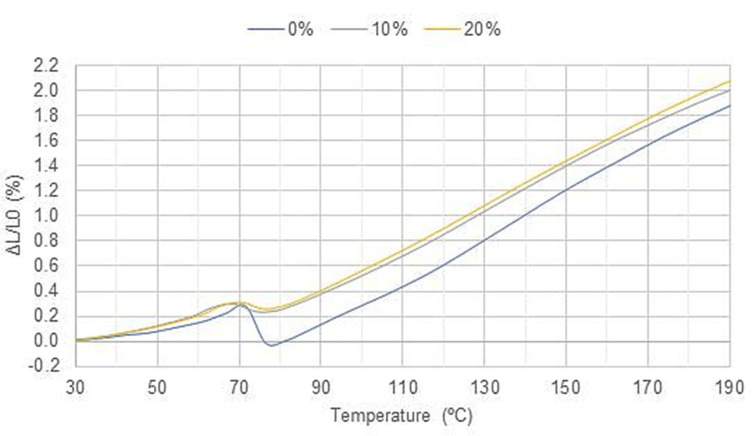
Results of CUP as a function of load.

**Figure 15:**
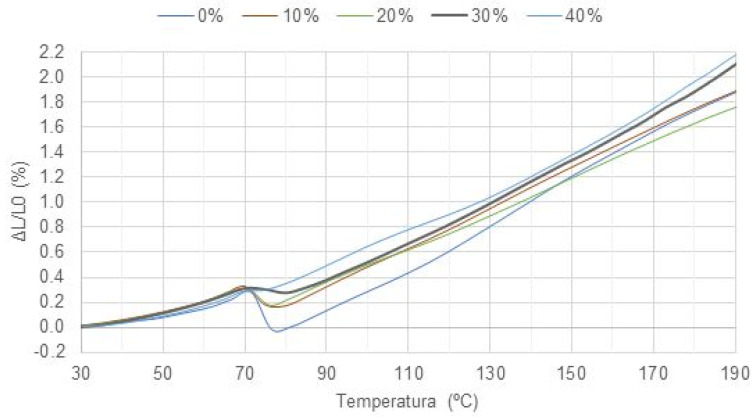
Results of CP as a function of load.

Figure 14 and Figure 15 show the results of the linear dilatometry of specimens made with processed and unprocessed particulates, respectively. It can be observed at the temperature of 70 °C there is a shrinkage that can be explained by the change in the mobility of polymer chains, which is associated with the glass transition of the material. As particles are inserted, a tendency to reduce such contraction can be observed, since they restrict the movement of polymeric chains. In the formulation with 40% volumetric fraction, for example, no contraction was observed, but a plateau, allowing this formulation to work with higher temperatures, close to melting point.

This behavior was expected, since particulates usually block the polymer chains movement, and also because if we have high amount of particulates, we do not have a polymer length of great extent and the epoxy resin tends to expand before melting. This is corroborated by researchers [46,47] who have shown that natural fillers have an expansion coefficient lower than resins, because of what is expected to reduce the retraction with the incorporation of natural particulates, such as coconut shell powders into polymeric resins.

Table 4 presents a numerical comparison of the main results obtained at linear dilatometry testing. Through the results, it is possible to observe that before retraction the load favors expansion, while during and after retraction it minimizes it.

When comparing the types of fillers with the same volumetric fraction, it is possible to observe that the processed particulates have a behavior closer to pure epoxy. This is attributed to the reduction in the average equivalent diameter and aspect ratio, since with smaller particles the movement of polymer chains is less obstructed. This implies that processed particulates tend to generate less internal stress.

## 7. Conclusions

In conclusion, the main points confirmed were:

- The processing with ball mill reduced the average size and aspect ratio of particulates;
- With smaller particulates, there is an improvement in the wettability and workability, allowing higher amounts of reinforcement to be worked with, increasing the impact and compression strengths;
- The reduction in size improves the dispersion and reduces the agglomeration of particulates;
- With a high amount, 40 vol%, of coconut husk powder, the work temperature for this formulation can be close to melting point, since the glass transition temperature is not observed at dilatometry;
- The finished surface is very smooth, enabling this new composite to be a potential anti-corrosive coating in the near future.

## Figures and Tables

**Table 1:** List of tests performed in this work.

Test	N° of Specimens	Parameters	Machine	Standard
Compression	5	Test speed: 2 mm/min Offset Yield: 0.2%	Universal Tensile Tester, Instron 5582	ASTM E228-17 [34]
Impact (Izod)	5	Impact Energy: 11 J Impact Velocity: 3.5 m/s	Pendulum Impact tester, Pantec XC-50	ASTM D256-10 [35]
Dilatometry	1	Temperature range: 300 to 460 k Heating rate: 2 K/min	Dilatometer Netzsch DIL 402 PC	ASTM D695-15 [36]

**Table 2:** Results obtained in the compression tests.

Filler (Volume)	Epoxy + Unprocessed Powder			Epoxy + Processed Powder		
	YS (MPa)	YM (MPa)	CS (MPa)	YS (MPa)	YM (MPa)	CS (MPa)
0%	67.6 ± 2.5	1891 ± 88	78.5 ± 1.6	67.6 ± 2.5	1891 ± 88	78.5 ± 1.6
10%	48.1 ± 7.5	1464 ± 140	57.9 ± 4.1	90.4 ± 1.4	1314 ± 32	90.4 ± 1.4
20%	17.5 ± 3.2	390 ± 77	20.1 ± 2.9	75.9 ± 3.3	1701 ± 22	82.7 ± 2.0
30%	-	-	-	63.7 ± 6.0	1734 ± 61	73.4 ± 1.3
40%	-	-	-	38.9 ± 6.1	853 ± 172	65.8 ± 8.7

**Table 3:** Results obtained in the Impact Izod tests.

Filler (Volume)	Epoxy + Unprocessed Powder		Epoxy + Processed Powder	
	Impact Strength (kJ/m^2^)	Notch Strength (J/m)	Impact Strength (kJ/m^2^)	Notch Strength (J/m)
0%	1.445 ± 0.075	14.32 ± 1.03	1.445 ± 0.075	14.32 ± 1.03
10%	0.995 ± 0.120	10.30 ± 1.31	1.454 ± 0.134	12.62 ± 1.16
20%	1.224 ± 0.271	12.81 ± 3.23	1.847 ± 0.153	19.85 ± 1.77
30%			1.675 ± 0.110	15.31 ± 1.38
40%			1.933 ± 0.153	19.13 ± 2.48

**Table 4:** Consolidation of the results obtained at linear dilatometry testing.

Formulation	Average Expansion Rate before Peak	Peak Contraction	Average Expansion Rate after Peak	Total Expansion (ΔL/L0)
**Pure epoxy**	0.0062/°C	22.70%	0.0179/°C	1.87%
**Epoxy + CUP 10vol%**	0.0065/°C	5.58%	0.0163/°C	2.00%
**Epoxy + CUP 20vol%**	0.0069/°C	4.96%	0.0168/°C	2.07%
**Epoxy + CP 10vol%**	0.0072/°C	13.42%	0.0157/°C	1.88%
**Epoxy + CP 20vol%**	0.0067/°C	11.08%	0.0141/°C	1.76%
**Epoxy + CP 30vol%**	0.0070/°C	3.60%	0.0160/°C	2.09%
**Epoxy + CP 40vol%**	0.0072/°C	0.00%	0.0162/°C	2.17%

## Data Availability

Not applicable.
